# Pathway-wide association study identifies five shared pathways associated with schizophrenia in three ancestral distinct populations

**DOI:** 10.1038/tp.2017.8

**Published:** 2017-02-21

**Authors:** C Liu, C A Bousman, C Pantelis, E Skafidas, D Zhang, W Yue, I P Everall

**Affiliations:** 1 Department of Psychiatry, Melbourne Neuropsychiatry Centre, The University of Melbourne and Melbourne Health, Carlton South, VIC, Australia; 2 Florey Institute of Neuroscience and Mental Health, The University of Melbourne, Parkville, VIC, Australia; 3 Department of General Practice, The University of Melbourne, Parkville, VIC, Australia; 4 Centre for Human Psychopharmacology, Swinburne University of Technology, Hawthorn, VIC, Australia; 5 Department of Electrical and Electronic Engineering, Centre for Neural Engineering (CfNE), University of Melbourne, Carlton South, VIC, Australia; 6 NorthWestern Mental Health, Melbourne, VIC, Australia; 7 Institute of Mental Health, The Sixth Hospital, Peking University, Beijing, China; 8 Key Laboratory of Mental Health, Ministry of Health & National Clinical Research Center for Mental Disorders (Peking University), Beijing, China; 9 Peking-Tsinghua Joint Center for Life Sciences/PKU-IDG/McGovern Institute for Brain Research, Peking University, Beijing, China

## Abstract

Genome-wide association studies have confirmed the polygenic nature of schizophrenia and suggest that there are hundreds or thousands of alleles associated with increased liability for the disorder. However, the generalizability of any one allelic marker of liability is remarkably low and has bred the notion that schizophrenia may be better conceptualized as a pathway(s) disorder. Here, we empirically tested this notion by conducting a pathway-wide association study (PWAS) encompassing 255 experimentally validated Kyoto Encyclopedia of Genes and Genomes (KEGG) pathways among 5033 individuals diagnosed with schizophrenia and 5332 unrelated healthy controls across three distinct ethnic populations; European-American (EA), African-American (AA) and Han Chinese (CH). We identified 103, 74 and 87 pathways associated with schizophrenia liability in the EA, CH and AA populations, respectively. About half of these pathways were uniquely associated with schizophrenia liability in each of the three populations. Five pathways (serotonergic synapse, ubiquitin mediated proteolysis, hedgehog signaling, adipocytokine signaling and renin secretion) were shared across all three populations and the single-nucleotide polymorphism sets representing these five pathways were enriched for single-nucleotide polymorphisms with regulatory function. Our findings provide empirical support for schizophrenia as a pathway disorder and suggest schizophrenia is not only a polygenic but likely also a poly-pathway disorder characterized by both genetic and pathway heterogeneity.

## Introduction

Schizophrenia is a severe psychiatric disorder characterized by significant genetic heterogeneity and is commonly referred to as a polygenic disorder.^1^ This polygenicity was most recently highlighted in the largest genome-wide association study (GWAS) of schizophrenia that identified 128 single-nucleotide polymorphisms (SNPs) at 108 loci associated with the disorder,^2^ although there are likely thousands of SNPs that contribute to its liability, many of which are population-specific. Thus, identifying schizophrenia-associated SNPs that are generalizable to diverse populations of people with the disorder is unlikely and suggests alternative approaches at identifying genetic markers of schizophrenia liability are needed.

One such approach is to examine SNPs within the biological pathway(s) in which they reside. Underlying this approach is the notion that schizophrenia is a pathway(s) disorder,^3^ whereby one or a number of SNPs within a pathway could result in an increased liability to schizophrenia by altering sensitivity to environmental insults and/or disruption of brain development. In the context of schizophrenia, this pathway approach has been applied in a variety of forms ranging from pathway clustering analysis,^4^ where SNPs in key genes within a single pathway are examined, to *post-hoc* pathway enrichment analyses of candidate SNP-sets using the SNP ratio test^5^ or bioinformatics resources (for example, Ingenuity Pathway Analysis).^6, 7, 8^ These approaches undoubtedly have a pathway focus but provide an incomplete examination of the compendium of known human biological pathways. Our primary aim was to conduct a comprehensive pathway-wide association study (PWAS) of schizophrenia. Here, we report results of that analysis in which we tested 255 biological pathway-based SNP-sets for their association and potential function in schizophrenia in three ancestral distinct populations.

## Materials and methods

### Data sources

GWAS data from individuals with schizophrenia (*n*=5033) and healthy controls (*n*=5332) across three distinct ethnic populations; European-American (EA) (2455 schizophrenia, 2826 controls), Han Chinese (CH) (1625 schizophrenia, 1527 controls) and African-American (AA) (953 schizophrenia, 979 controls) were obtained (Table 1; Supplementary Table S1). EA and AA GWAS data were collected by the Genetic Association Information Network (GAIN) and nonGAIN projects and were obtained through the database of Genotypes and Phenotypes (dbGaP, phs000021.v1.p1 and phs000167.v1.p1)^9^ with ethics approval by The University of Melbourne Human Ethics Committee (#1340723). CH GWAS data were collected from multiple collaborating hospitals included in the Chinese Schizophrenia Collaboration Group (see Supplementary Materials for details).^10^ For the EA and CH cohorts two independent datasets were available. One was used as a discovery dataset (EA: 1215 schizophrenia cases and 1442 healthy control subjects; CH: 1159 schizophrenia cases and 1089 healthy control subjects) and the other a validation dataset (EA: 1240 schizophrenia and 1384 controls; CH: 466 schizophrenia and 438 controls).

### Quality control and population stratification

We adhered to a previously published quality control protocol^11^ with the exception of procedures related to identity by descent and population stratification (see Supplementary Materials for details). Population stratification was mitigated using spatial ancestry analysis (SPA).^12^ The SPA European model was used for the analysis of the EA data, whereas the SPA worldwide model was used for the AA and CH datasets. We used all available genotypes to calculate the geographic coordinates of latitude and longitude and set inclusion boundaries (Supplementary Figure S1). The final sample sizes and SNPs available for analysis following quality control are presented in Table 1 (see Supplementary Table S1 for details on the number of individuals and SNPs removed at each quality control step).

### Mapping SNPs to genes and pathways

SNPs surviving quality control were mapped to gene loci using the annotation provided by the National Center for Biotechnology Information (see Supplementary Methods for details). Genes were then mapped to pathways curated by the Kyoto Encyclopedia of Genes and Genomes (KEGG, Release 76.0, 1 October, 2015),^13^ which includes 301 human pathways from six main categories (metabolism, genetic Information processing, environmental information processing, cellular processes, organismal systems and human diseases). A mega KEGG pathway (metabolic pathways, hsa01100) that encompasses several other pathways was excluded, leaving 300 pathways available for further analysis.

### Pathway-wide association analysis

**Figure 1:**
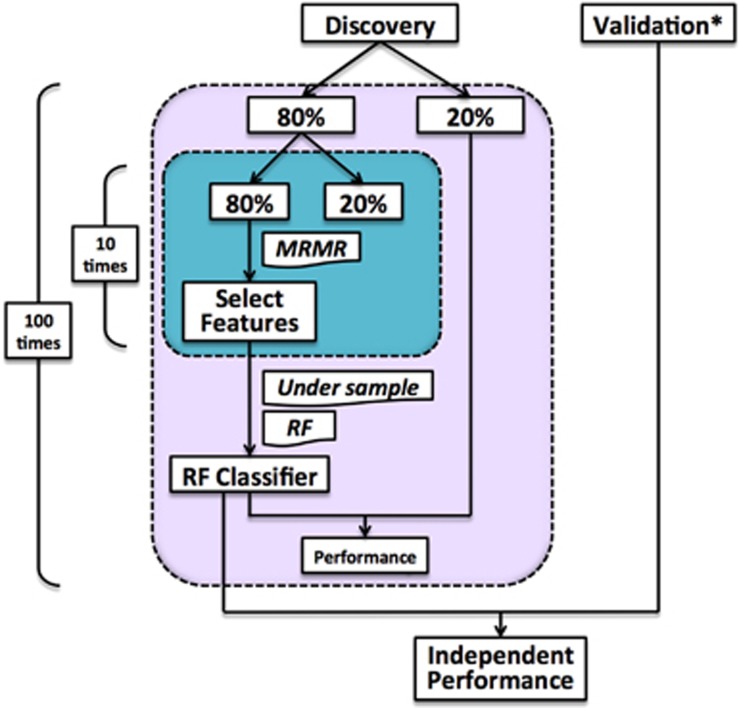
Pathway-wide association analysis pipeline. *Only the European American population and the Han Chinese population have independent validation dataset. MRMR, maximum relevance minimum redundancy; RF, random forest.

The analysis pipeline used to assess each of the 300 KEGG pathways for their association with schizophrenia is depicted in Figure 1, evolving from our previously published pathway analysis pipeline.^14^ For each pathway the discovery dataset for the EA and CH cohorts as well as the single dataset available for the AA cohort were randomly split (maintaining the case:control ratio of the full dataset) 100 times into two subsets, a SNP (that is, feature) selection set (80% of the participants) and a test set (20% of participants). Within each SNP selection set, 80% of participants were randomly selected 10 times (maintaining the case:control ratio of the full dataset) and the resulting subsets were subjected to the maximum relevance minimum redundancy (mRmR) feature selection procedure (blue box, Figure 1).^15^ The mRmR procedure was chosen as an alternative to *P*-value-based feature selection procedures that are dependent on sample size and do not necessarily result in feature sets that maximize relevance and minimize redundancy (that is, increase mutual information; see Supplementary Materials and Supplementary Figure S2 for details and a comparison of the two feature selection methods in our datasets). This procedure resulted in 300 SNP sets, one for each of the KEGG pathways (Supplementary Table S2). Among these 300 SNP sets, 45 sets containing less than two features (SNPs) at one or more of the 100 iterations were excluded from further analysis, as our algorithm requires two or more features to fit a model.

The 255 SNP sets were then used to build 255 classifiers, one for each KEGG pathway, via a random forest algorithm with default parameters (R package: ‘randomForest') using 80% of the discovery dataset followed by testing of the classifiers in the remaining 20% of the discovery dataset. To address inherent imbalances in the case:control ratios of our datasets, under-sampling of the majority class (cases or controls) of each dataset was employed before running the random forest algorithm, as this strategy has previously been shown to be useful for classification in the presence of imbalanced classes.^16, 17^

To assess the overall performance of each pathway classifier, the random forest model derived from the selected SNPs for each pathway within the 80% discovery set was applied to the 20% test set as well as the independent validation dataset, with the exception of the AA cohort for which an independent validation dataset was not available. In addition, within the 20% test set and independent validation dataset, case–control labels of all individuals were randomly permuted and the random forest model for each pathway derived from the 80% discovery set was applied, with the exception of the AA cohort. Point estimates and 95% confidence intervals for five performance metrics (accuracy, sensitivity, specificity, area under the receiver operating characteristic curve (AUC) and odds ratio (OR)) were calculated for each of the pathways using the independent validation dataset for EA and CH cohorts, and the 20% test set for the AA cohort. *P*-values for each of the 255 pathways were generated by comparing the mean OR (based on 100 iterations) from the independent (validation for AA) dataset with the corresponding mean OR from the permutation dataset using a *t*-test. The Benjamini–Hochberg (BH) procedure was used to adjust for multiple comparisons.^18^ Furthermore, using the independent validation dataset for the EA and CH cohorts, we calculated the Nagelkerke *R*^2^ (see Supplementary Materials for details) to estimate the variance in schizophrenia liability explained by each of the 255 SNP sets used to construct the pathway classifiers. The complete annotated computer script used to conduct the pathway-wide association analysis is available upon request.

To further evaluate our pathway analysis pipeline, we selected 129 previously identified gene ontology (GO) pathways associated with schizophrenia and applied our pipeline to each of the 129 pathways in all three populations.

### Functional analysis of SNPs in candidate pathways

To assess whether the mRmR feature selection approach was capable of selecting informative features and to evaluate the potential functional relevance of selected features, we utilized the brain expression quantitative trait loci (eQTLs) dataset obtained from the genotype-tissue expression (GTEx) portal v6.0,^19^ as well as the functional annotation information obtained from the RegulomeDB, a database that annotates SNPs with known and predicted regulatory elements.^20^ We hypothesized that the selected features with greater appearance rates within significant schizophrenia liability pathways would be enriched for functional SNPs compared with SNP sets derived from non-significant pathways.

#### eQTL analysis

SNP sets representing pathways associated with schizophrenia in all three cohorts were further assessed as potential *cis*-eQTLs using genotype and gene expression data derived from human post-mortem frontal cortex (Brodmann area 9) of 92 donors included in the GTEx portal.^19^ For each of the three cohorts, SNPs within each of our candidate pathways was assigned an appearance rate based on the number of times (out of 100 iterations) the SNP represented the candidate pathway during our feature selection procedure (that is, mRmR) described above. SNPs were then grouped into quartiles based on their appearance rate (that is, 0–25% 26–50% 51–75% and 76–100%) and the proportion of SNPs within each quartile associated (alpha threshold=0.05) with expression of its corresponding gene was calculated. For comparison, the same analysis was conducted on 152, 181 and 168 non-significant pathways in the EA, CH and AA population, respectively. A one-sample *t*-test was used to determine if the proportion of eQTLs observed in our overlapping pathway SNP sets differed from the SNP sets derived from non-significant pathways.

#### Regulome analysis

To investigate the potential functional significance of selected features beyond eQTLs, including DNA–protein interaction (TF-binding motif, DNase footprint) and DNA–RNA interaction (microRNA-binding motif, long non-coding RNA), we utilized the RegulomeDB (http://www.regulomedb.org).^20^ Similar to the eQTL analysis, SNPs were grouped into quartiles based on their appearance rate and a weighted Regulome score was computed for each quartile group as well as SNP sets derived from non-significant pathways. A one-sample *t*-test was used to determine if the weighted Regulome score in our candidate SNP sets differed from SNP sets derived from non-significant pathways (see Supplementary Materials for details).

## Results

### Pathway-wide association analysis

**Figure 2:**
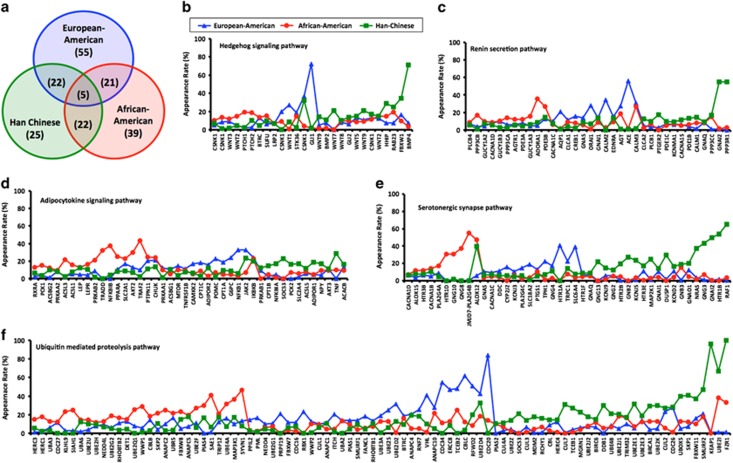
(**a**) Venn diagram showing the degree of overlap in pathways identified as a liability for schizophrenia across the three ancestrally distinct populations. (**b–f**) Relative contribution (measured by appearance rate in our feature selection procedure) of genes within the five common schizophrenia liability pathways by ancestry. Only genes with appearance rates summing to 20% or greater across the three populations are shown for each pathway (see Supplementary Table S6 for appearance rates of all single-nucleotide polymorphisms (SNPs) in each of the five pathways). Each gene's appearance rate is normalized to the number of haplotype-tagging SNPs (threshold *R*^2^=0.50) within the gene.

Of the 255 pathways examined, 103, 74 and 87 pathways were significantly associated with schizophrenia liability in the EA, CH and AA cohorts, respectively (Supplementary Figure S3; Supplementary Table S3–5). Examination of the overlap between the cohorts showed 55, 25 and 39 pathways were uniquely associated with schizophrenia liability in the EA, CH and AA cohorts, respectively (Figure 2a). Five pathways (serotonergic synapse, ubiquitin mediated proteolysis, hedgehog signaling, adipocytokine signaling and renin secretion) were shared across all three cohorts (Figure 2a). However, the relative contribution of the SNPs and genes within these five pathways differed considerably by ancestry (Figures 2b–e; Supplementary Table S6) and a small subset of genes were shared across two or three common pathways (Supplementary Figure S4). Sensitivity, specificity, AUC, accuracy and ORs were modest for each of the five pathways and no pathway explained more than one percent (*R*^2^=0.03–0.57%) of the variance in the liability to schizophrenia (Table 2). Combining the selected features from the five shared pathways had minimal impact on the variance explained (*R*^2^=0.31–0.57%), although when features from all significant pathways were assessed the variance explained ranged from 0.66% in the EA cohort to 2.46% in the CH cohort. Furthermore, among the 129 previously identified schizophrenia-associated GO pathways our analysis pipeline replicated 45, 20 and 56 of these pathways in the EA, CH and AA populations (Supplementary Table S7).

### Functional analysis of SNPs in candidate pathways

**Figure 3:**
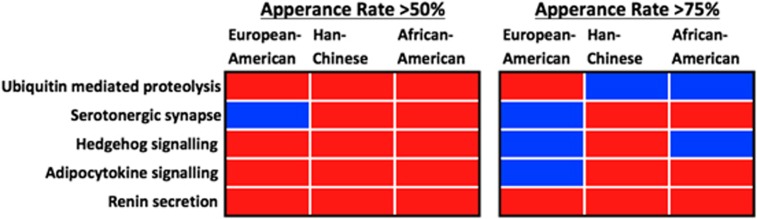
Summary of the functional analysis performed on single-nucleotide polymorphisms (SNPs) with an appearance rate of >50% and >75% during feature selection in the five shared pathways across the three populations. Red boxes indicate the proportion of functional SNPs (based on GTEx or RegulomeDB data) was significantly greater compared with SNP sets derived from non-significant pathways within that population. Blue boxes indicate the proportion of functional SNPs was significantly lower compared with SNP sets derived from non-significant pathways within that population.

Analysis of SNPs selected to represent the five pathways that overlapped in the three populations showed SNPs with greater appearance rates had a greater probability of being an eQTL or having some other regulatory function (Figure 3; Supplementary Figures S5 and S6). SNPs that appeared >50% of the time during our feature selection procedure were more likely to be functional compared with 100 random SNP sets of equal size, with the exception of the serotonergic synapse SNPs in EAs. Likewise, SNPs with appearance rates >75% also had a higher probability to be functional, although for six of the pathway-population pairs (Figure 3) our feature selection procedure did not identify SNP sets enriched for functional SNPs.

## Discussion

The notion that schizophrenia is a pathway disease has only recently been proposed^3^ and as such empirical testing of this notion is limited. We conducted a PWAS of schizophrenia in three ancestrally distinct cohorts. We found evidence of pathway heterogeneity in schizophrenia liability, identified five pathways conferring liability across populations and showed that the SNP sets representing these five pathways were enriched for SNPs with regulatory functions.

Pathway heterogeneity has only recently been discussed in the context of schizophrenia^4^ but has been well characterized in other diseases such as cancer.^21, 22^ Pathway heterogeneity builds on and encompasses the concept of genetic heterogeneity in that it postulates a disorder is a result of one or more perturbations in one or more of a multiple number of pathways. Supporting this notion, we found that nearly half (47%) of the pathways we tested were uniquely associated with schizophrenia liability in only one of the three populations we examined—raising the possibility that schizophrenia is not only a polygenic but also a poly-pathway disorder. In fact, all pathways had an OR<1.35, suggesting multiple pathways of small effect collectively contribute to schizophrenia liability.

Furthermore, our results suggest disruption of certain pathways may be necessary (but perhaps not sufficient) for the development of schizophrenia across populations. About one-fourth (27%) of the pathways we tested were associated with liability to schizophrenia in two or more of the populations, among which five pathways were associated with schizophrenia liability in all three cohorts. These pathways included the serotonergic synapse, ubiquitin mediated proteolysis, hedgehog signaling, renin secretion and adipocytokine signaling, all of which have been implicated in schizophrenia and/or related phenotypes.

A number of post-mortem, functional neuroimaging and peripheral biomarker studies have implicated the serotonergic system in the pathophysiology of schizophrenia (for review see: ref. 23) and many atypical antipsychotic agents (for example, clozapine, olanzapine) are potent serotonin receptor 2A antagonists.^24^ Thus, identification of the serotonergic synapse pathway in the current study is perhaps not surprising. In fact, the largest schizophrenia GWAS to date found SNPs in three genes (*CACNA1C*, *ITPR3* and *CYP2D6*) within the serotonergic synapse pathway reached GWAS significance (*P*<5 × 10^−8^) and SNPs in another 17 genes within this pathway were nominally significant (*P*<1 × 10^−5^).^2^ Furthermore, a recent gene-set enrichment analysis of the SZGene database^25^ identified 24 pathways significantly enriched for schizophrenia candidate genes among which the serotonin receptor signaling pathway was ranked second.^7^

The ubiquitin mediated proteolysis pathway (UPP), a critical system for the removal of damaged/toxic proteins in the cell, has been shown to be dysregulated at the transcript^26, 27, 28, 29^ and protein levels^30^ in both peripheral and central tissue among individuals with schizophrenia. In addition, peripheral transcript levels within the UPP have been associated with positive symptom severity^31^ and a recent copy number variant meta-analysis in schizophrenia, autism and intellectual disability revealed that two ubiquitin-related gene-ontologies were highly enriched with schizophrenia-associated copy number variants.^32^ Furthermore, animal studies have suggested that UPP has an important role in regulating synaptic growth and neural circuits,^33, 34^ and demand on the UPP at pre-synaptic and post-synaptic terminals may in part link dysfunction of the UPP to increased schizophrenia liability.^35^

The hedgehog signaling pathway is a key regulator of oligodendrocyte production,^36, 37^ dopaminergic neuron development,^38^ and promotes brain expression of *disc1*, a candidate gene in schizophrenia.^39^ The pathway's most well characterized ligand, sonic hedgehog, regulates the generation of functional synaptic contacts,^40, 41^ and is abundant in the adult human central nervous system.^42, 43^ Furthermore, hedgehog signaling interacts with the UPP^44^ and has been implicated in the ‘two-hit' hypothesis of schizophrenia by which disruption of the pathway during brain development primes the central nervous system for a pathologic response to a second hit in later life.^45^

The renin secretion pathway is typically associated with regulation of arterial blood pressure, thirst and thermoregulation via the kidney secreted enzyme renin and its interaction with the renin–angiotensin–aldosterone system. Epidemiological studies have reported up to 25% of schizophrenia have polydipsia (excessive thirst)^46^ and in general exhibit dysregulation of body temperature.^47^ Rodent studies have demonstrated renin is also synthesized in the brain^48^ and has considerable effects on anxiety-related behaviors and cognition (for example, memory).^49^ In the brain, renin is proposed to enzymatically process angiotensinogen to angiotensin, which is then further processed by angiotensin-converting enzyme (ACE).^50^ ACE activity has been shown to modulate dopamine turnover^51^ and abnormal levels of ACE in cerebrospinal fluid have been reported in individuals with schizophrenia,^52, 53^ albeit potential neurotropic and length of illness effects have been noted.^54, 55^ The interaction between angiotensin II (AT II), a neuropeptide substrate for ACE, and central dopamine has also been associated with schizophrenia.^56, 57^ Moreover, numerous genetic studies suggested polymorphisms in *ACE* are associated with susceptibility to schizophrenia and major depression.^58, 59, 60, 61, 62^

The adipocytokine signaling pathway is a collective destination of cytokines secreted by the adipose tissue. Since the first adipocytokine leptin was discovered in 1994,^63^ hundreds of adipocytokines have been found, such as adiponentin, tumor necrosis factor-alpha and members of the interleukin family. Increased expression of tumor necrosis factor-alpha and a number of interleukins have recently been proposed as markers of schizophrenia in brain^64^ and blood.^65^ Furthermore, adipocytokines are recognized not only as regulators of energy metabolism, but also as factors that may be associated with mental disorders. Decreased serum levels of adiponectin have been identified in major depressive disorder and schizophrenia,^66, 67, 68^ and serum levels of leptin correlate with less severe positive symptoms in schizophrenia patients^69^ and may regulate the mesolimbic dopamine system.^70^

Despite the novelty and many strengths of our study, our findings should be interpreted in the context of several limitations. First, the detection of population differences in schizophrenia liability at the pathway level may, in part, be a result of sampling, allelic frequency and/or linkage disequilibrium differences across the populations studied. These potential confounding factors may also explain why we only identified five overlapping pathways rather than the expected 10 overlaps given the number of significant pathways identified in each population. Although we attempted to reduce these confounding influences by selecting features independently in each population using an mRmR approach, complete restraint of these confounds is not possible and as such our results should be interpreted with caution. Second, an independent validation dataset was not available for the AA population and as such all estimates were based on the 20% holdout dataset derived from the discovery dataset. This may have resulted in over-estimation of associations within this population, although the random forest algorithm we employed internally mitigates this potential bias via the out-of-bag error estimate mechanism.^71^ Third, the dopamine hypothesis of schizophrenia is an enduring, widely accepted, idea but among the three populations we studied the dopaminergic synapse pathway was only a significant liability pathway in the EA (OR=1.108, 95% CI=1.088–1.127; B-H *P*=8.18E−13) and CH (OR=1.054, 95% CI 1.027-1.08; B-H *P*=0.050) populations. Although, we failed to detect this pathway in the AA population, the expected trend was evident (OR=1.056, 95% CI=1.011–1.102; B-H *P*=0.429) and is likely a result of the smaller sample size available for this population. Fourth, our analysis did not look at potential clinical subtypes of schizophrenia based on symptom profiles, despite recognition that schizophrenia is a broader spectrum disorder including a range of symptoms. A PWAS of clinical subtypes may lead to stronger associations by reducing the noise associated with the broad schizophrenia phenotype. However, the clinical symptom data available for this study was inconsistent or minimal across the three cohorts inhibiting such an analysis. Fifth, SNPs eligible for inclusion in our analysis were limited to those that were within a gene using a narrow ‘5 and 3' intergenic window (2 and 0.5 kbp, respectively). Intergenic SNPs are known to play functional regulatory roles on genes^72^ and as such exclusion of more distal intergenic SNPs may have biased our results. In the most recent schizophrenia GWAS,^2^ 45% (57) of the 128 SNPs identified were intergenic but 52% (30) of these intergenic SNPs are in linkage disequilibrium (*R*^2^⩾0.50) with one or more SNPs within a gene according to our intergenic window. Thus, our intergenic window was capable of capturing a majority (79%, *n*=101) of the 128 SNPs, suggesting the bias conferred by our SNP inclusion criteria are likely modest. Finally, our feature selection procedure (mRmR) resulted in the loss of many pathways containing smaller SNP pools. In total, 45 pathways cataloged within KEGG were not included in our pathway association study as the number of SNPs selected to represent these pathways was fewer than the required number of SNPs (that is, 2) to run our random forest algorithm.

In conclusion, our results empirically support the notion that schizophrenia is a pathway disorder and further suggest that there is a considerable amount of pathway heterogeneity within and across different ethnic populations. We also identified five pathways that may serve as harbors of genotypic markers for schizophrenia across populations. However, future application of our pathway-wide association approach in larger cohorts as well as among ethnic groups not examined here are required before firm conclusions can be drawn.

## Figures and Tables

**Table 1:** Sample size and SNPs available for analysis following quality control procedures

*Ancestry*	*Cases*			*Controls*			*Platform*	*SNPs*	*Source*
	N	*Age (s.d.)*	*Male/female*	N	*Age (s.d.)*	*Male/female*			
*European-American*									
Discovery	972	43.71 (11.29)	681/291	1248	50.61 (17.05)	570/678	Affymetrix 6.0	691 822	GAIN
Replication	879	42.26 (11.93)	609/270	1132	49.91 (15.77)	569/563	Affymetrix 6.0	691 822	Non-GAIN
									
*Han Chinese*									
Discovery	1125	35.97 (7.82)	555/570	1034	36.60 (10.35)	476/558	Illumina Zhonghua 8	800 509	CSCG
Replication	454	36.48 (7.98)	262/192	411	36.40 (8.16)	187/224	Illumina Zhonghua 8	800 509	CSCG
									
African-American	896	43.30 (10.12)	558/338	906	45.16 (13.03)	344/562	Affymetrix 6.0	818 941	GAIN

Abbreviations: CSCG, Chinese Schizophrenia Collaboration Group; GAIN, Genetic Association Information Network; SNPs, single-nucleotide polymorphisms.

**Table 2:** Performance metrics for the four common pathways across the three ethnically distinct cohorts

*Point estimate (95% CI)*	*Ubiquitin-mediated proteolysis*			*Serotonergic synapse*			*Hedgehog signaling*			*Adipocytokine signaling pathway*			*Renin secretion*		
	*EA*	*AA*	*CH*	*EA*	*AA*	*CH*	*EA*	*AA*	*CH*	*EA*	*AA*	*CH*	*EA*	*AA*	*CH*
Sensitivity	0.530 (0.526–0.534)	0.515 (0.508–0.522)	0.519 (0.514–0.523)	0.522 (0.519–0.525)	0.506 (0.499–0.513)	0.491 (0.486–0.496)	0.513 (0.509–0.516)	0.510 (0.503–0.517)	0.495 (0.491–0.500)	0.520 (0.517–0.523)	0.515 (0.508–0.522)	0.491 (0.486–0.496)	0.524 (0.521–0.527)	0.508 (0.501–0.516)	0.490 (0.485–0.494)
Specificity	0.490 (0.487–0.493)	0.505 (0.498–0.512)	0.506 (0.502–0.511)	0.491 (0.487–0.494)	0.508 (0.501–0.515)	0.524 (0.519–0.529)	0.504 (0.500–0.508)	0.504 (0.496–0.512)	0.522 (0.518–0.527)	0.505 (0.501–0.508)	0.507 (0.499–0.514)	0.525 (0.520–0.529)	0.492 (0.488–0.495)	0.505 (0.497–0.513)	0.524 (0.520–0.529)
AUC	0.515 (0.513–0.517)	0.518 (0.514–0.523)	0.520 (0.517–0.523)	0.508 (0.506–0.510)	0.509 (0.505–0.514)	0.512 (0.509–0.515)	0.511 (0.509–0.513)	0.511 (0.505–0.516)	0.511 (0.508–0.515)	0.517 (0.514–0.519)	0.514 (0.509–0.518)	0.509 (0.506–0.512)	0.511 (0.509–0.514)	0.508 (0.503–0.514)	0.511 (0.508–0.514)
Accuracy	0.512 (0.510–0.515)	0.510 (0.506–0.515)	0.512 (0.510–0.515)	0.509 (0.507–0.510)	0.507 (0.503–0.511)	0.508 (0.505–0.511)	0.509 (0.507–0.511)	0.507 (0.502–0.512)	0.509 (0.506–0.512)	0.513 (0.511–0.515)	0.511 (0.506–0.515)	0.509 (0.506–0.511)	0.510 (0.508–0.512)	0.509 (0.502–0.512)	0.508 (0.505–0.511)
Odds ratio	1.087 (1.069–1.104)	1.104 (1.065–1.144)	1.113 (1.088–1.138)	1.057 (1.041–1.073)	1.072 (1.038–1.107)	1.070 (1.043–1.098)	1.073 (1.056–1.091)	1.077 (1.036–1.117)	1.080 (1.054–1.105)	1.108 (1.09–1.127)	1.109 (1.069–1.150)	1.072 (1.046–1.098)	1.069 (1.053–1.086)	1.080 (1.036–1.124)	1.065 (1.041–1.090)
Adjusted-*P*	1.07 × 10^−7^	0.0139	0.00242	0.000285	0.0492	0.00420	1.55 × 10^−7^	0.00615	2.41 × 10^−5^	2.89 × 10^−14^	0.0103	0.0121	3.03 × 10^−8^	0.0139	0.00597
Nagelkerke *R*^2^^a^	0.0005	—	0.0057	0.0017	—	0.0015	0.0003	—	0.0033	0.0011	—	0.0005	0.0010	—	0.0012

Abbreviations: AA, African-American cohort; AUC, area under the curve; CH, Han Chinese cohort; EA, European-American cohort.

a Refer to the Supplementary Materials for details.
