# Supplementary material for: Pathway-wide association study identifies five shared pathways associated with schizophrenia in three ancestral distinct populations
Source: Transl Psychiatry. 2017 Feb 21;7(2):e1037–. doi: 10.1038/tp.2017.8 (PMC5438037; doi:10.1038/tp.2017.8)
Supplement: Supplementary Information [file tp20178x1.docx]

**SUPPLEMENTARY METHODS**

**Pathway-wide association study identifies five shared pathways associated with schizophrenia in three ancestral distinct populations**

**Table of Contents** __

# Han Chinese recruitment 2

# Quality control of GWA studies 2

# Mapping SNPs to genes procedures 2

Comparison of feature selection methods 3

Nagelkerke R^2^ analysis 3

Regulome analysis 3

**Supplementary Figures** __

**Figure S1**. Spatial ancestry analysis plots by cohort 4

**Figure S2.** Feature selection method comparison 5

**Figure S3.** Heatmap summarizing results of a pathway-wide association analysis 7

**Figure S4.** Shared genes and their relative contribution in five common pathways 8

**Figure S5.** Functional analysis results by RegulomeDB annotation system 9

**Figure S6**. Functional analysis results by eQTL in frontal cortex tissue 10

**References 11**

**Supplementary methods**

# Han Chinese recruitment

The Han Chinese population is characterised by “north-south” population structure.[^1^](#_ENREF_1)^,^ [^2^](#_ENREF_2) In this study, both cases and controls in the Han Chinese GWAS dataset were recruited from the Northern China regions by three research centres of the Chinese Schizophrenia Collaboration Group (Peking University Sixth Hospital, Huilongguan Hospital, Beijing Anding Hospital). The North Han Chinese subjects in the discovery dataset were recruited from Tianjin, Hebei, Liaoning, Shaanxi, Henan, Heilongjiang and Jilin by the research centres at Peking University Sixth Hospital and Huilongguan Hospital. The North Han Chinese subjects in independent dataset were recruited from Shandong, Jiangsu, Liaoning and Jiangxi by the research centre at Beijing Anding Hospital. Consensus diagnoses were made by at least two experienced psychiatrists using the Structured Clinical Interview for the Diagnostic and Statistical Manual of Mental Disorders IV. No subjects had severe medical complications or other psychiatric disorders. All control individuals were clinically determined to be free of psychiatric disorders or family history of such disorders, including first-, second- and third-degree relatives. All participants provided written informed consent. The Han Chinese GWAS was approved by the Institutional Ethical Committee of each hospital.

**Quality control of GWA studies**

We first excluded samples with an undefined phenotype from each of five GWAS, and also excluded 130 individuals from the European-American validation set as these participants were identified as non-European ancestry by SPA.[^3^](#_ENREF_3) Then each of these five datasets was processed based on a standardized, previously published quality control protocol[^4^](#_ENREF_4) with the exception of procedures related to IBD and population stratification. In detail, an individual was removed from further analysis if any of the following was present:

1. Self-reported sex discordance with sex chromosomes.
2. Missing > 5% of SNPs or an anomalous heterozygosity rate identified (beyond Mean[het] ± 3 × s.d.[het] ).
3. Had a PI_HAT of > 0.10 with more than 10% of the study sample.[^5^](#_ENREF_5)
4. One of a pair of blood relatives (PI_HAT > 0.185).

SNPs were excluded from the dataset if any of the following was present:

1. Significant different missing rate between case and control groups.
2. Deviation from Hardy-Weinberg equilibrium ( *p* <= 1e-05 )
3. Missing rate > 5%.
4. Minor allele frequency < 0.01.

The SPA program was then applied to identify the ancestry population as well as whether case and control groups were genetically matched. After the QC-cleaned GWAS data was obtained, SNPs located within sex chromosome and mitochondrial chromosome and SNPs that weren’t common in discovery GWAS set and corresponding validation set were excluded from the final dataset for subsequent analysis.

# Mapping SNPs to genes and pathways

Two tables (SNPContigLocusid and SNPChrPosOnRef) were downloaded from dbSNP142 FTP site.[^6^](#_ENREF_6) The information of biological pathways was obtained from KEGG Release 76.[^7^](#_ENREF_7) Genotyped SNPs in each GWAS were assigned to a human gene if the SNP was located within 2kb of the 5’ and 0.5kb of the 3’ boundary of a gene according to the dbSNP142 database. SNPs within genes were then mapped to a biological pathway if their host gene was involved in a pathway within the KEGG database.

**Feature selection methods**

As shown in Figure 1, the GWAS datasets were randomly split into an 80% selection set and a 20% test set. This random partition process was repeated 100 times for each of 300 KEGG pathways. Within each selection set, 80% of the participants were randomly selected 10 times and then delivered to the mRmR feature selection procedure to extract the SNP set with maximum relevance and minimum redundancy.[^8^](#_ENREF_8) In detail, we selected the recommended MID (Mutual Information Difference) scheme and “Categorical” option, which is consistent with the format of our genotyping data. The mRmR scores were calculated for each SNP in the KEGG pathway and an average mRmR score across the subsets from 10 iterations was calculated for each SNP in each pathway. SNPs with an average mRmR score greater than zero were retained for further analysis as a score < zero equates to no increase in mutual information.

The mRmR procedure was used as an alternative method to traditional p-value strategy. To compare these two SNP-selection methods, we generated the Manhattan plots for the common pathways in three populations and highlighted selected features in red and enlarged dots (Supplementary Figures S2).

**Nagelkerke R^2^ analysis**

To assess how much variance in schizophrenia liability the five overlapping significant pathways explained, we conducted Nagelkerke R^2^ analysis[^9^](#_ENREF_9) in European and Chinese populations, as both of them had independent validation GWAS datasets. First, we computed a polygenic risk score (PRS) for each individual for each pathway by using the ‘--score’ function in Plink software[^10^](#_ENREF_10) with its default commands. *W_score_* was used as the score for each of selected SNPs in the pathway. *W_score_* was computed with the following formula:

$$W_{score}=\left( {SNP}_{or}-1 \right)\times{SNP}_{ap}$$

Where, ${SNP}_{or}$ was the odds ratio of each SNPs reference allele, and ${SNP}_{ap}$ was the appearance rate based on the number of times the SNP appeared (out of 100 iterations) in our feature selection procedure. The Nagelkerke R^2^ was computed by comparison of a model including PRS and covariates (PRS + gender + PCA1 + PCA2) to a covariate only model (gender + PCA1 + PCA2).

**Regulome Analysis**

The category score of Regulome DB[^11^](#_ENREF_11) annotated by dbSNP132 were obtained from the Regulome website (http://www.regulomedb.org/downloads). The sub groups of category 1 (from 1a to 1f) were integrated and the number 1 was assigned to these sub groups as their category score. The same process was applied to Regulome category 2 and 3. Selected features in pathways divided into quartiles based on their appearance rate and a weighted Regulome score ($W_{rs}$) were computed for each quartile group as well as SNP sets from non-significant pathways:

$$W_{rs}= \sum(7-category score)$$

The score represented the functional score of SNPs assigned by RegulomeDB. One-sample t test was applied to $W_{rs}$ of quartile groups against the reference panel taken from non-significant pathways.

**Supplementary Figures**

(a)
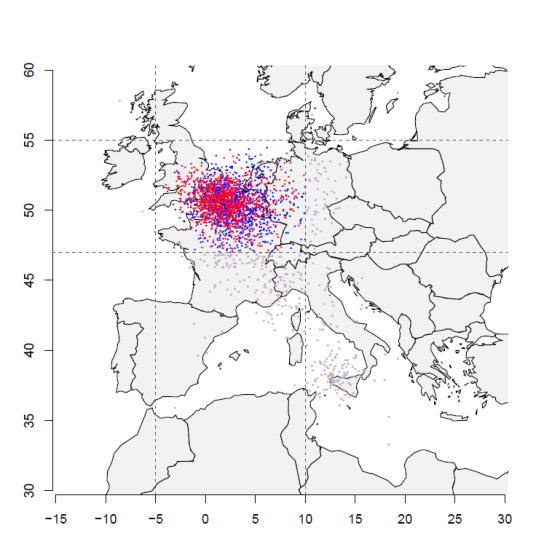
 (b)
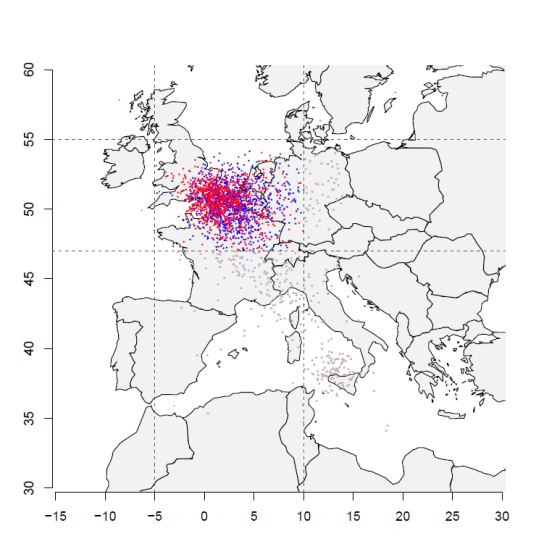


(c)
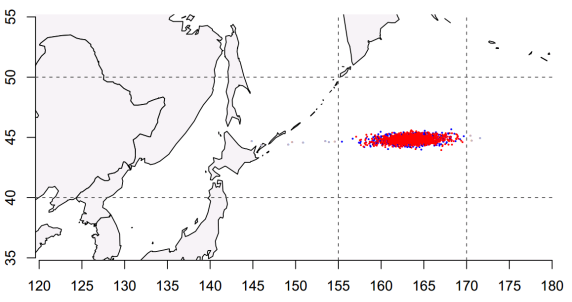
(d)
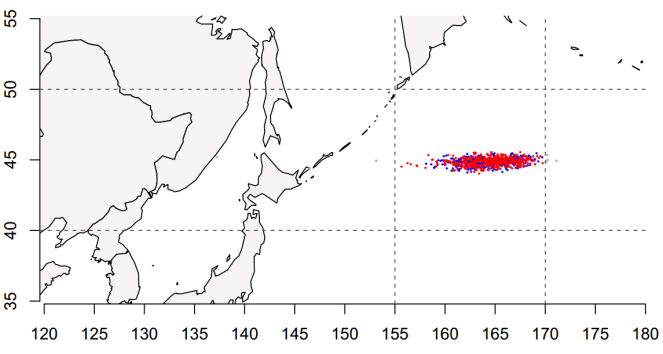


(e)
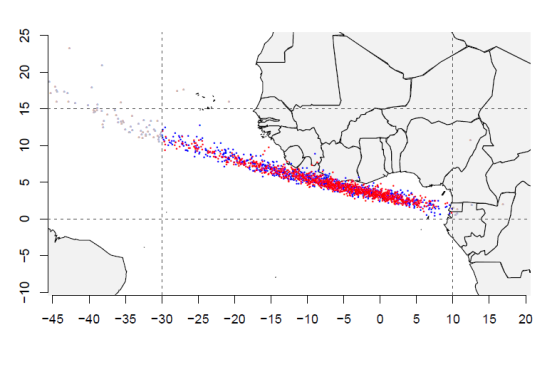


**Supplementary Figure S1**. Ancestry of GAIN, nonGAIN and Han Chinese GWAS cohort (a-e). Filter criteria used in the study limited to the individual located within N47-55 and W5-E10 for European discovery (a) and independent validation dataset (b), N40-50 and E155-170 for Han Chinese discovery (c) and independent validation dataset (d), N0-15 and W30-0 for African America population(e).

**
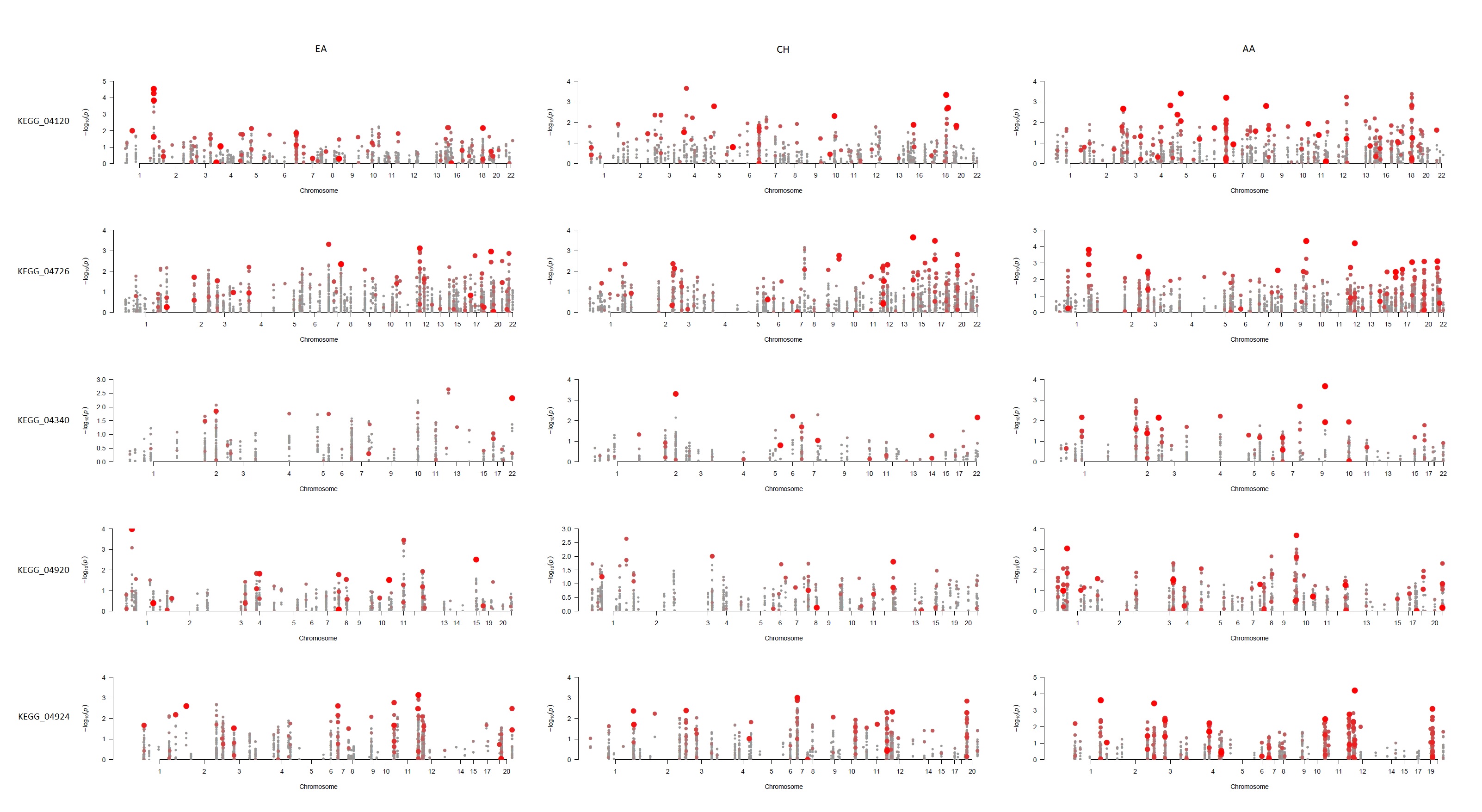
**

**Supplementary Figure S2.** Comparison of selected (red) and unselected (grey) SNPs within the five overlapping liability pathways in the European-American (EA), Chinese-Han (CH), and African-American (AA) cohorts. The size of the red dots relates to the number of times the SNP appeared during the 100 iterations of our feature selection procedure. Kegg_04120 = ubiquitin mediated proteolysis; Kegg_04726 = serotonergic synapse; Kegg_04340 = hedgehog signaling; Kegg_04920 = adipocytokine signalling; Kegg_04924 = renin secretion.

Supplementary Figure S3: Please see the uploaded original JPEG-format file.

**
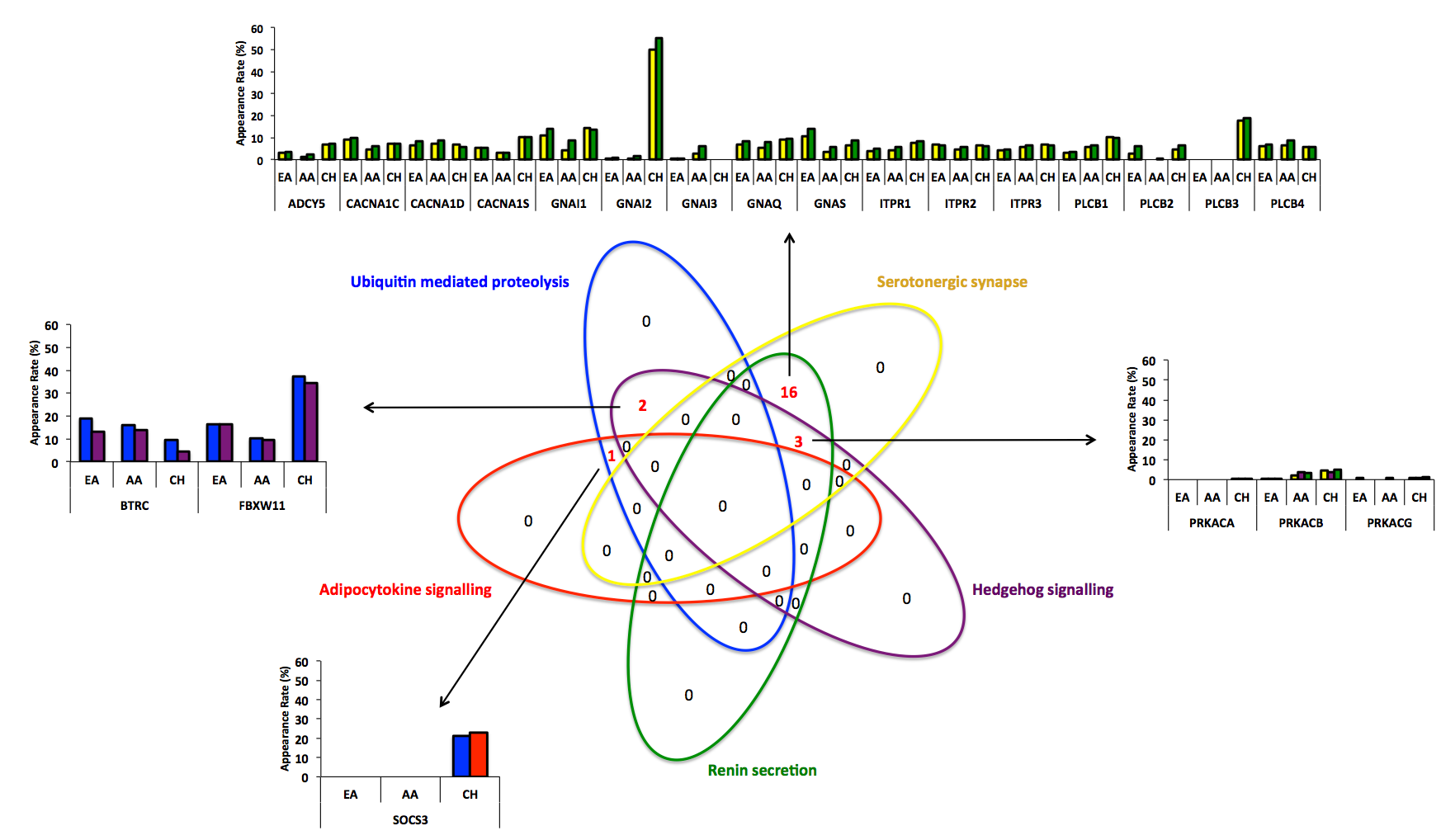
**

**Supplementary Figure S4**: Shared genes and their relative contribution (measured by appearance rate in our feature selection procedure) across the five overlapping pathways. The numbers within each ellipse and combination of ellipses represent the number of genes within each pathway and shared genes between the pathways, respectively. Bar colors correspond to the ellipse color of the pathway they represent. Each gene’s appearance rate is normalised to the number of haplotype-tagging SNPs (threshold r-squared = 0.50) within the gene.


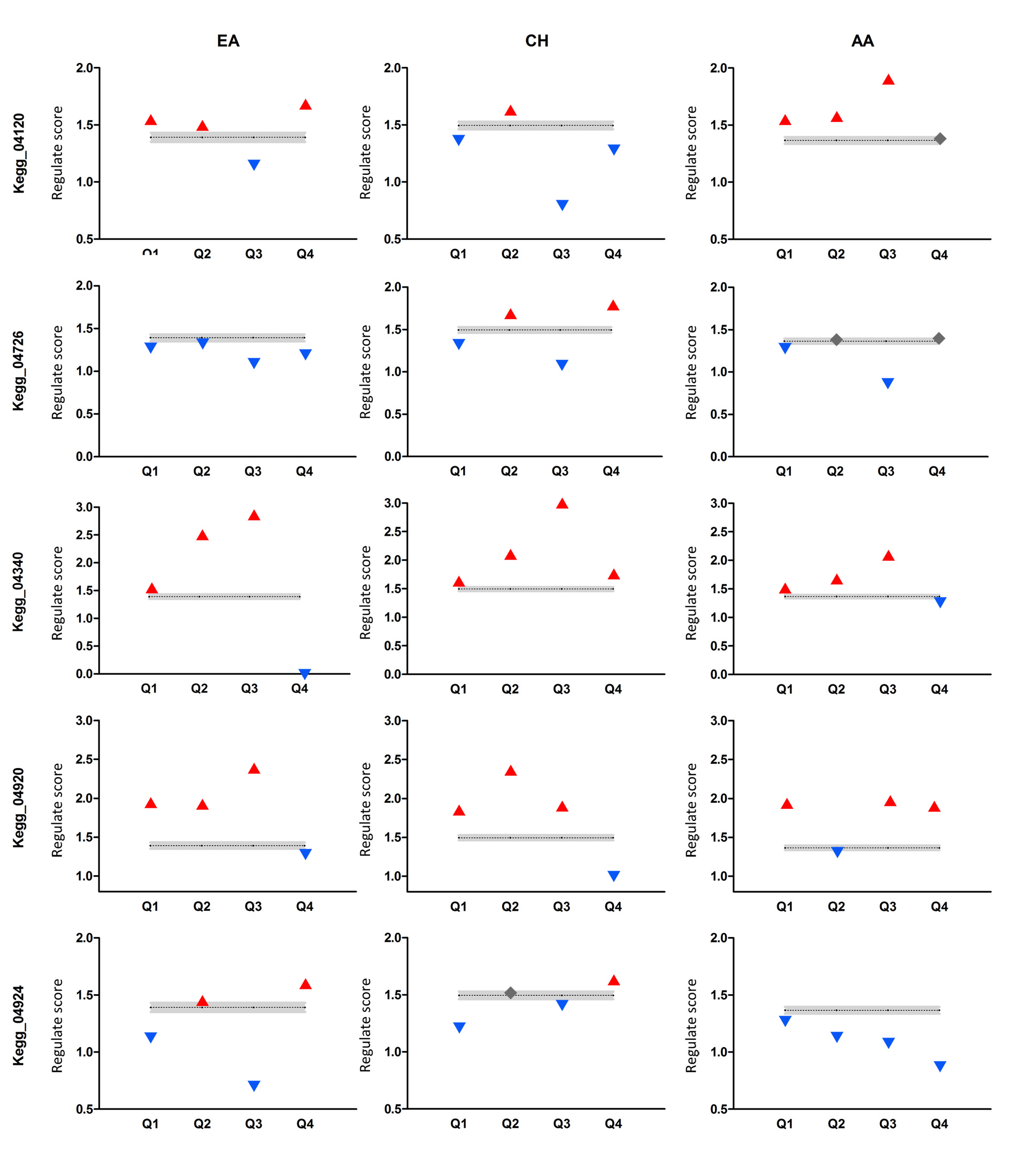


**Supplementary Figure S5.** Results of functional analysis using the RegulomeDB annotation system by pathway and ancestry. Analysis was performed on quartile groups of SNPs based on their appearance rate (i.e. 0 – 25%; 26 – 50%; 51 – 75%; 76 -100%) during our 100 iteration feature selection procedure. The grey shadow region represents the mean value and 95% confidence interval of non-significant pathway SNP sets. Red triangles denote significantly (p < 0.05) greater Regulome score relative to non-significant pathway SNP sets. Blue inverted triangles denote significantly (p < 0.05) lower Regulome score relative to the non-significant pathway SNP sets. Grey diamond denotes no difference in Regulome score relative to the non-significant pathway SNP sets. EA = European-American; AA = African-American; CH = Han-Chinese. Kegg_04120 = ubiquitin mediated proteolysis; Kegg_04726 = serotonergic synapse; Kegg_04340 = hedgehog signaling; Kegg_04920 = adipocytokine signalling; Kegg_04924 = renin secretion.

**
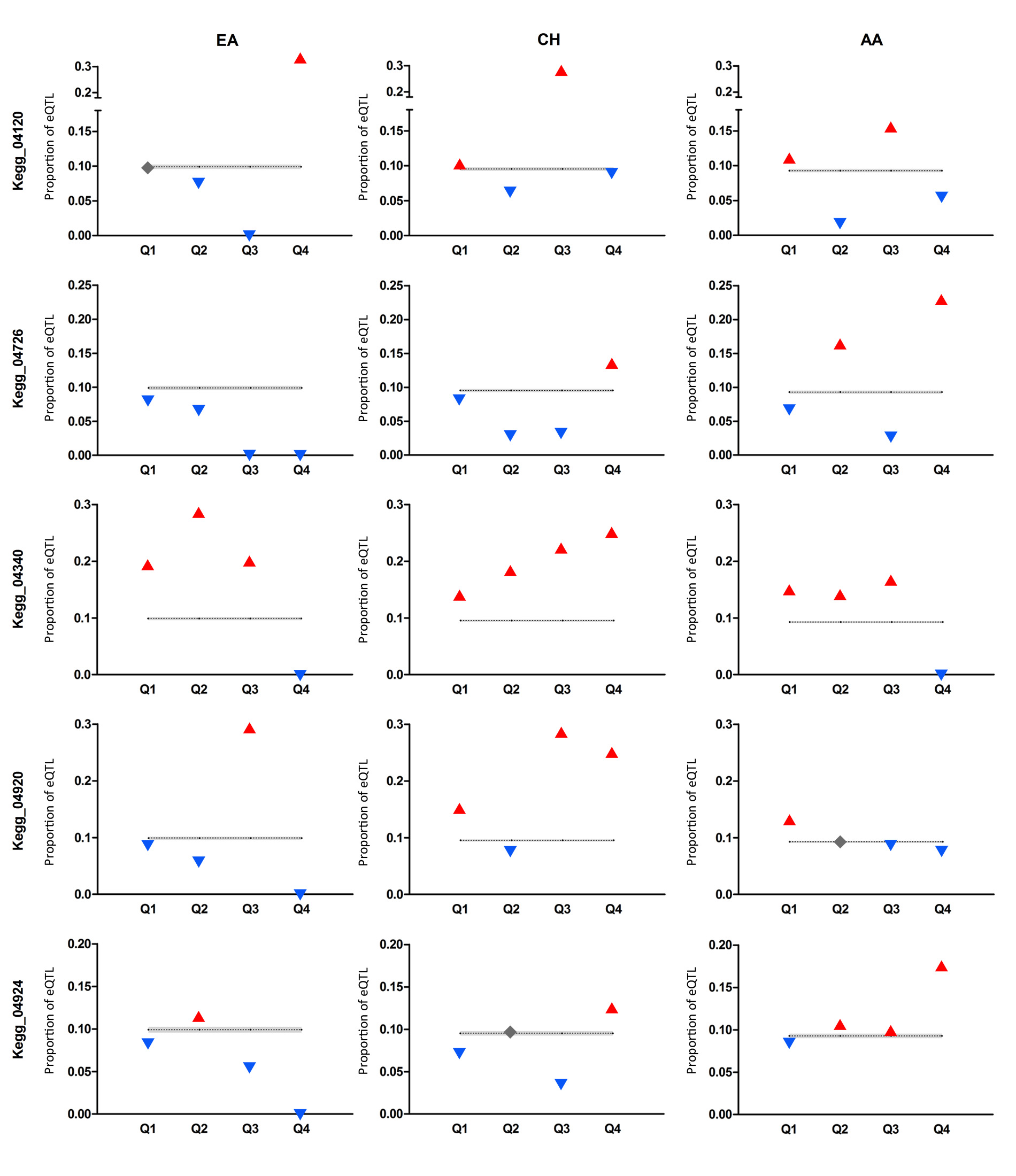
**

**Supplementary Figure S6.** Results of functional analysis by eQTL in frontal cortex by pathway and ancestry. Analysis was performed on quartile groups of SNPs based on their appearance rate (i.e. 0 – 25%; 26 – 50%; 51 – 75%; 76 -100%) during our 100 iteration feature selection procedure. The grey shadow region represents the mean value and 95% confidence interval of non-significant pathway SNP sets. Red triangles denote significantly (p < 0.05) greater proportion of eQTL relative to the non-significant pathway SNP sets. Blue inverted triangles denote significantly (p < 0.05) lower proportion of eQTL relative to the non-significant pathway SNP sets. Grey diamond denotes no difference in the proportion of eQTL relative to the non-significant pathway SNP sets. EA = European-American; AA = African-American; CH = Han-Chinese. Kegg_04120 = ubiquitin mediated proteolysis; Kegg_04726 = serotonergic synapse; Kegg_04340 = hedgehog signaling; Kegg_04920 = adipocytokine signalling; Kegg_04924 = renin secretion.

**REFERENCES**

1. Xu S, Yin X, Li S, Jin W, Lou H, Yang L *et al.* Genomic dissection of population substructure of Han Chinese and its implication in association studies. *Am J Hum Genet* 2009; **85**(6)**:** 762-774.

2. Chen J, Zheng H, Bei JX, Sun L, Jia WH, Li T *et al.* Genetic structure of the Han Chinese population revealed by genome-wide SNP variation. *Am J Hum Genet* 2009; **85**(6)**:** 775-785.

3. Yang WY, Novembre J, Eskin E, Halperin E. A model-based approach for analysis of spatial structure in genetic data. *Nat Genet* 2012; **44**(6)**:** 725-731.

4. Anderson CA, Pettersson FH, Clarke GM, Cardon LR, Morris AP, Zondervan KT. Data quality control in genetic case-control association studies. *Nat Protoc* 2010; **5**(9)**:** 1564-1573.

5. Turner S, Armstrong LL, Bradford Y, Carlson CS, Crawford DC, Crenshaw AT *et al.* Quality control procedures for genome-wide association studies. *Curr Protoc Hum Genet* 2011; **Chapter 1:** Unit1 19.

6. Sherry ST, Ward MH, Kholodov M, Baker J, Phan L, Smigielski EM *et al.* dbSNP: the NCBI database of genetic variation. *Nucleic Acids Res* 2001; **29**(1)**:** 308-311.

7. Kanehisa M, Goto S. KEGG: kyoto encyclopedia of genes and genomes. *Nucleic Acids Res* 2000; **28**(1)**:** 27-30.

8. Peng H, Long F, Ding C. Feature selection based on mutual information: criteria of max-dependency, max-relevance, and min-redundancy. *IEEE Trans Pattern Anal Mach Intell* 2005; **27**(8)**:** 1226-1238.

9. Nagelkerke NJ. A note on a general definition of the coefficient of determination. *Biometrika* 1991; **78**(3)**:** 691-692.

10. Purcell S, Neale B, Todd-Brown K, Thomas L, Ferreira MA, Bender D *et al.* PLINK: a tool set for whole-genome association and population-based linkage analyses. *Am J Hum Genet* 2007; **81**(3)**:** 559-575.

11. Boyle AP, Hong EL, Hariharan M, Cheng Y, Schaub MA, Kasowski M *et al.* Annotation of functional variation in personal genomes using RegulomeDB. *Genome Res* 2012; **22**(9)**:** 1790-1797.
